# The impact of a community-based HIV and sexual reproductive health program on sexual and healthcare-seeking behaviors of female entertainment workers in Cambodia

**DOI:** 10.1186/s12879-015-0954-4

**Published:** 2015-06-06

**Authors:** Siyan Yi, Sovannary Tuot, Pheak Chhoun, Carinne Brody, Khimuy Tith, Sopheap Oum

**Affiliations:** Research Center, No. 33, Street 71, P.O Box 2311, Phnom Penh, Khana Cambodia; Public Health Program, College of Education and Health Sciences, Touro University-California, California, USA

**Keywords:** Female entertainment workers, Community-based intervention, SAHACOM, Sexual reproductive health, HIV, STIs, Healthcare-seeking behaviors, Cambodia

## Abstract

**Background:**

In Cambodia, despite great successes in the fight against HIV, challenges remain to eliminating new HIV infections and addressing sexual reproductive health (SRH) issues in key populations including female entertainment workers (FEWs). To address these issues, the Sustainable Action against HIV and AIDS in Communities (SAHACOM) project has been implemented since late 2009 using a community-based approach to integrate HIV and SRH services. This study evaluates the impact of the SAHACOM on sexual and healthcare-seeking behaviors among FEWs in Cambodia.

**Methods:**

A midterm and endpoint comparison design was utilized. Midterm data were collected in early 2012, and endpoint data were collected in early 2014. A two-stage cluster sampling method was used to randomly select 450 women at midterm and 556 women at endpoint for face-to-face interviews.

**Results:**

Compared to women at midterm, women at endpoint were significantly less likely to report having sexual intercourse in exchange for money or gifts in the past three months (OR = 2.1, 95 % CI = 1.6-2.7). The average number of commercial sexual partners in the past three months also decreased significantly from 5.5 (SD = 13.3) at midterm to 3.6 (SD = 13.9) at endpoint (*p* = 0.03). However, women at endpoint were significantly less likely to report always using condom when having sexual intercourse with clients in exchange for money or gifts (OR = 2.6, 95 % CI = 1.5-4.5). Regarding sexually transmitted infections (STIs), women at endpoint were significantly less likely to report having an STI symptom in the past three months (OR = 1.8, 95 % CI = 1.4-2.3) and more likely to seek treatment for the most recent STI symptom (OR = 1.6, 95 % CI = 1.1-1.9). Furthermore, women at endpoint were significantly more likely to be currently using a contraceptive method (OR = 1.4, 95 % CI = 1.1-1.8) and less likely to report having an induced abortion (OR = 1.4, 95 % CI = 1.1-1.7) during the time working as a FEW.

**Conclusions:**

The overall findings of the study indicate that the SAHACOM is effective in reducing sexual risk behaviors and improving the access to SRH care services among FEWs in Cambodia. However, several unfavorable findings merit attention.

## Background

Cambodia is one of the few countries in the world that has reversed HIV epidemic from being generalized to concentrated, meaning that it is now confined mainly to individuals who engage in high-risk behaviors [1]. In 2013, the National Center for HIV/AIDS, Dermatology, and STD (NCHADS) estimated that HIV prevalence among general adult population aged 15 to 49 was 0.6 %, reflecting a significant decline from the peak of 2.0 % in 1998 [2]. This success was widely attributed to the 100 % condom use program that led to a significant increase in condom use as well as a decline in the frequency of commercial sex and an increase in access to HIV voluntary confidential counseling and testing (VCCT) and antiretroviral therapy (ART) [3, 4]. As a result, Cambodia was presented with a Millennium Development Goals (MDG) Award at the MDG Summit in 2010 [5]. This award represents a global recognition of the outstanding national leadership, commitment, and progress towards achievement of Goal 6 – halting and reversing the spread of HIV [5].

Despite these great achievements, eliminating new HIV infections remains a challenge. The epidemic is now concentrated in high-risk populations, including female entertainment workers (FEWs) [2]. In Cambodia, FEWs refer to women employed in the entertainment industry which include beer promoters, karaoke singers, massage workers, hostesses in restaurants, or women working in other entertainment establishments such as bars, night clubs, or beer gardens. These women are considered at risk regardless of their involvement in direct or indirect sex work [6].

Recent studies have indicated particularly high HIV prevalence among different groups of FEWs such as brothel-based (17.4 %) and street-based (37.3 %) sex workers as well as women working in other entertainment establishments (9.8 %) [7]. Sexually transmitted infections (STIs) [7, 8] and induced abortion [9] are also very common among these women. Consistent condom use, particularly with regular and non-paying partners, remains very low [9, 10] and access to sexual reproductive health (SRH) care services is limited [9]. According to the most recent national behavioral sentinel surveillance, prevalence of consistent condom use with commercial and non-commercial partners among FEWs were 81 % and 36 %, respectively [11]. Moreover, 38 % of FEWs reported having at least one STI symptom, while 30 % had never used STI care services, and 70 % had been tested for HIV in the past 12 months [11]. Regarding SRH, 70 % of FEWs reported currently using a contraceptive method, and 77 % reported having at least one abortion during the time working as a FEW [11].

For prevention of a resurgence of the epidemic, intervention programs must be tailored to the current needs of these vulnerable young women. To address these issues, KHANA – a leading non-governmental organization (NGO) providing community-based integrated HIV prevention, care, and support services in Cambodia – has implemented the Sustainable Action against HIV and AIDS in Communities (SAHACOM) project since October 2009. The goals of SAHACOM for FEWs are to: (1) increase condom use with commercial and non-commercial partners; (2) decrease number of commercial partners; (3) increase rates of regular HIV and STI testing; (4) increase use of modern contraceptive methods; (5) decrease induced abortion rates; and (6) increase treatment-seeking for STI symptoms.

The SAHACOM program utilizes a community-based approach to empower and create ownership in communities [12, 13]. Through this model, community support volunteers, peer facilitators, and outreach workers are utilized to implement the project activities. The project activities include: (1) outreach sessions to FEWs at their workplaces led by FEWs trained as outreach workers who use behavior change communication techniques to promote condom use, HIV/STI testing, family planning, and reproductive health services; (2) community health volunteers offering venue-based counseling and finger-prick HIV/syphilis testing to FEWs as well as case management and referrals for care and treatment at health facilities; and (3) drop-in vocational training centers for FEWs seeking new professions that provides trainings on tailoring, make-up artistry, and hairdressing. The drop-in centers also provide places where FEWs can meet and provide peer support in a safe environment. By September 2013, the SAHACOM had reached 6,311 FEWs with individual or small group preventive and care interventions.

The SAHACOM is among a few comprehensive community-based interventions providing a full package of HIV and SRH services to key populations in developing countries. It is necessary to document lessons learned from this project and disseminate them in the literature. We therefore conducted this study to measure the impact of the SAHACOM on sexual and healthcare-seeking behaviors of FEWs in Cambodia.

## Methods

A midterm and an endpoint survey were conducted in 2012 and 2014, respectively. Similar methods, tools, and procedures were used for both surveys. Details of the surveys have been published elsewhere [14–16].

### Study population and sampling

The z test for a two-sample comparison of proportions was employed to detect a change of 10 % of sexual and healthcare-seeking behavior indicators such as consistent condom use with regular partners and utilization of VCCT services. A power of 80 % with 95 % confidence interval (CI) and design effect of 2 for compensation of cluster effect was set for the sample size calculation. As a result, the total minimum required sample size was approximately 410 women. Adjusted for incomplete data of 10 %, the final minimum sample size required for the surveys was approximately 460.

The study was conducted in Phnom Penh and Siem Reap. We excluded other provinces because the population size of FEWs under the SAHACOM was too small at the time when the midterm survey was conducted. Furthermore, the total number of FEWs in these two provinces represented more than 70 % of the total coverage of the integrated care and prevention and 100 % of the total coverage of the focused prevention. In total, 41 entertainment establishments in Phnom Penh and 13 in Siem Reap were covered by the SAHACOM with a total number of FEWs of 5,404 and 1,445, respectively exposed to the intervention.

The sample size was proportionally allocated to the size of FEWs under the SAHACOM in each province. A two-stage cluster sampling method was used to select the study samples. Communes were used as sampling units, and the number of entertainment establishments in each selected commune was decided based upon the number of FEWs in each commune. In order to be included in the study, an entertainment establishment must have at least 20 FEWs. We also considered other factors when deciding whether to include an entertainment establishment such as convenience for data collection and duration of the project implementation in the commune. The participants were then randomly selected from the entertainment establishments.

A FEW would be included in the study if she was: (1) 18 years or older; (2) Khmer speaking; (3) working for an entertainment establishment under the coverage of the SAHACOM; (4) sexually active in the past 12 months; (5) able to provide consent to participate in the study; and (6) able to present themselves on the day of the data collection. FEWs who were mentally and/or physically too sick to participate were excluded from the study. In total, 450 FEWs at midterm and 556 FEWs at endpoint were included in this analysis.

### Data collection trainings and procedures

All interviewers and field supervisors were trained for two days on questionnaire and data collection methods, and one day was allocated for tool pretesting and reflection. The purpose of the training was to make sure that all research team members understood the procedures and followed the standardized guidelines in the same manner. The training covered necessary skills including interview techniques, confidentiality, questionnaire administration, and quality control. Regular review sessions with interviewers were conducted during the survey period to review progress and communicate any problems or issues occurring during the data collection. Coordination and administration were arranged by KHANA’s implementing partners. Subjects were interviewed face-to-face with an estimated time for each interview of approximately 30 min.

### Questionnaire development and measurement

The questionnaire was developed using standardized tools adapted from previous studies in the same populations [17, 18], the most recent Cambodia Demographic and Health Survey [19], as well as from other studies in Cambodia [20–24]. The questionnaire was initially developed in English and then translated into Khmer, the national language of Cambodia. Another translator back-translated it into English to ensure that the “content and spirit” of every original item were maintained. Clear instructions and explanations were addressed to avoid any confusion during the interviews.

Before constructing the final questionnaire, a pilot study was conducted among a random sample of 20 participants to ensure that wording and contents were culturally suitable, acceptable, and clearly understandable for the study participants. We made necessary modifications based on feedback from the pilot study and comments from researchers and practitioners working in the areas of HIV and SRH in Cambodia.

Variables included socioeconomic characteristics, sexual behaviors, SRH, healthcare seeking behaviors, HIV testing, and HIV education. We collected information regarding their sexual experiences, involvement in commercial sex, and condom use behaviors when having sexual intercourse with unpaid or regular partners as well as with commercial partners. Regarding SRH, we asked about their experiences and healthcare-seeking behaviors for the most recent STI symptoms, pregnancy, induced abortion, and contraceptive use.

### Ethical considerations

Participation in this study was voluntary which was made clear to the participants both before and during the consenting process. Informed consent was obtained from each participant after a detailed description of the study objectives and procedures was provided. Privacy of the respondents was strictly protected, and confidentiality was ensured by removing all personal identifiers from the survey questionnaires. The questionnaires and data collected from the respondents were kept under the responsibility of KHANA’s Research Center. The study protocol was approved by the National Ethics Committee for Health Research, Ministry of Health, Cambodia (No. 082NECHR).

### Data entry and analyses

Data were coded and entered into a computerized database using Epi Data version 3 (Odense, Denmark). Double data entry was performed to minimize entry errors. Chi-square test or Fisher’s Exact test was used for categorical variables and paired Student’s *t*-test for continuous variables to compare socio-economic characteristics of respondents and outcome indicators at midterm and endpoint to detect changes throughout the SAHACOM lifespan. Two-sided *p*-values of less than 0.05 were regarded as statistically significant. We used SPSS version 20.0 (IBM Corporation, New York, USA) for all data analyses.

## Results

### Socio-economic characteristics

Table 1 shows the comparisons of socio-economic characteristics of women at midterm and endpoint. In general, the characteristics of the respondents were similar. However, women at endpoint were significantly more likely to live with friends (OR = 1.5, 95 % CI = 1.1-2.3) or by themselves (OR = 1.8, 95 % CI = 1.2-2.6). Regarding their workplaces, women at endpoint were significantly less likely to work in a beer garden (OR = 1.9, 95 % CI = 1.1-3.4) but more likely to work in other entertainment establishments such as hairdresser’s, barber’s, street base, etc. (OR = 6.7, 95 % CI = 3.0-14.8).

**Table 1:** Comparisons of socio-economic characteristics of FEWs at midterm (*n* = 450) and endpoint (*n* = 556)

Characteristics	Midterm	Endpoint	
	*n* (%)	*n* (%)	OR (95 % CI)
Mean age (in years, ± SD)	25.7 ± 5.1	26.4 ± 5.5	0.42
Marital Status			
Never married	161 (33.1)	183 (32.9)	1.1 (0.9-1.4)
Married and living together	145 (32.2)	190 (34.2)	1.1 (0.8-1.4)
Divorced, separated, or widowed	156 (34.7)	183 (32.9)	1.1 (0.8-1.3)
Mean years of formal education completed	6.2 ± 3.2	6.1 ± 3.1	0.89
Mean monthly income (in USD)	227 ± 193	231 ± 221	0.68
Place of employment in the past 12 months			
Karaoke bar	241 (53.6)	261 (46.9)	1.1 (0.9-1.4)
Restaurant	132 (29.2)	163 (29.3)	1.1 (0.8-1.3)
Massage parlor	43 (9.6)	56 (10.1)	1.1 (0.7-1.6)
Beer garden	27 (6.0)	18 (3.2)	1.9 (1.1-3.4)
Other (hairdresser’s, barber’s, street based, etc.)	7 (1.6)	58 (10.4)	6.7 (3.0-14.8)
Mean duration of living in the current city (in months)	140 ± 124	142 ± 126	0.71
Mean duration working as FEWs (in months)	25.7 ± 9.1	30 ± 8.4	0.08
Mean duration working for the current job (in months)	15.8 ± 5.4	18.1 ± 5.2	0.16

Abbreviations: *CI* confidence interval, *FEWs* female entertainment workers, *OR* odds ratio, *SD* standard deviation

Values are number (%) with OR (95 % CI) for categorical variables and mean ± SD with p-value for continuous variables

### Sexual behaviors

Comparisons of sexual behaviors among FEWs at midterm and endpoint are shown in Table 2. Women at endpoint were significantly less likely to report having sexual intercourse in exchange for money or gifts in the past three months (OR = 2.1, 95 % CI = 1.6-2.7). The average number of commercial sexual partners in the past three months also decreased significantly from 5.5 (SD = 13.3) at midterm to 3.6 (SD = 13.9) at endpoint (*p* = 0.03). Among respondents who reported involvement in commercial sexual intercourse in the past three months, women at endpoint were significantly less likely to report always using condom when having sexual intercourse in exchange for money or gifts (OR = 2.6, 95 % CI = 1.5-4.5). The proportion of respondents who reported always using condom with unpaid or regular partners remained low and was not appreciably different in a comparison between women at midterm and endpoint (OR = 1.1, 95 % CI = 0.8-1.7).

**Table 2:** Comparisons of sexual behaviors among FEWs at midterm (*n* = 450) and endpoint (*n* = 556)

Sexual behaviors	Midterm	Endpoint	
	*n* (%)	*n* (%)	OR (95 % CI)
Had sexual intercourse with regular partners (past 3 months)	220 (48.1)	205 (46.9)	1.2 (0.7-1.9)
Always used condom with regular partners (past 3 months)	75 (34.1)	64 (31.4)	1.1 (0.8-1.7)
Had sexual intercourse with clients (past 3 months)	167 (28.1)	124 (22.5)	2.1 (1.6-2.7)
Mean number of commercial partners (past 3 months)	5.5 ± 13.3	3.6 ± 13.9	0.03
Always used condom with commercial partners (past 3 months)	103 (85.8)	100 (80.6)	2.6 (1.5-4.5)
Able to find condom when needed it (past 3 months)	347 (77.1)	426 (76.6)	1.1 (0.8-1.7)

Abbreviations: *CI* confidence interval, *FEW* female entertainment workers, *OR* odds ratio, *SD* standard deviation

Values are number (%) with OR (95 % CI) for categorical variables and mean ± SD with p-value for continuous variables

### STIs and healthcare-seeking behaviors

As shown in Table 3, compared to women at midterm, women at endpoint were significantly less likely to report having at least one STI symptom in the past three months (OR = 1.8, 95 % CI = 1.4-2.3). Of those who had experienced an STI symptom in the past three months, only 52.6 % of women at midterm sought treatment for the most recent STI symptoms compared to 71.4 % among women at endpoint (OR = 1.6, 95 % CI = 1.1-1.9). Regarding health facilities of choice, women at endpoint were significantly more likely to receive the first care and treatment for the most recent STI symptoms at an NGO clinic (OR = 1.9, 95 % CI = 1.1-3.3) and less likely to seek care and treatment from a private pharmacy (OR = 1.7, 95 % CI = 1.1-3.5) compared to women at midterm.

**Table 3:** Comparisons of STI symptoms and care seeking behaviors among FEWs at midterm (*n* = 450) and endpoint (*n* = 556)

STI symptoms and care seeking behaviors	Midterm	Endpoint	
	*n* (%)	*n* (%)	OR (95 % CI)
Had an STI symptom in the past 3 months	196 (43.6)	135 (24.3)	1.8 (1.4-2.3)
Sought care and treatment for the STI symptoms	103 (52.6)	95 (71.4)	1.6 (1.1-1.9)
Facility where the first care and treatment for the most recent STI symptom was obtained			
Public health center/clinic/hospital	16 (15.7)	14 (13.3)	1.2 (0.5-2.5)
NGO clinic	46 (44.7)	62 (59.0)	1.9 (1.1-3.3)
Pharmacy	23 (22.1)	15 (14.3)	1.7 (1.1-3.5)
Other (private clinic, traditional healer)	18 (17.5)	14 (13.4)	1.4 (0.6-2.9)
Person who advised to seek STI care and treatment			
Myself	29 (27.8)	38 (39.2)	1.4 (0.8-2.5)
Relatives/friends/colleagues	8 (7.6)	16 (18.6)	2.2 (0.9-5.3)
Peer educator/outreach worker/NGO staff	55 (53.8)	33 (34.0)	1.5 (0.9-2.6)
Other (partner, relative, manager)	11 (10.8)	8 (8.2)	1.3 (0.5-3.3)

Abbreviations: *CI* confidence interval, *FEWs* female entertainment workers, *NGO* non-governmental organization, *OR* odds ratio, *SD* standard deviation, *STIs* sexually transmitted infections

Values are number (%) with OR (95 % CI) for categorical variables and mean ± SD with p-value for continuous variables

### HIV testing and counseling

As shown in Table 4, 74.2 % of women at midterm and 63.9 % of women at endpoint reported having been tested for HIV in the past six months (OR = 1.3, 95 % CI = 1.1-1.6). Women at endpoint were significantly more likely to get tested for HIV at a community- or peer-initiated testing and counseling (OR = 1.7, 95 % CI = 1.4-2.2) and at a private laboratory, clinic, or hospital (OR = 1.9, 95 % CI = 1.3-2.7) but less likely to get tested at a VCCT center (OR = 3.3, 95 % CI = 2.3-4.9). Regarding referrals for HIV testing, women at endpoint were significantly less likely to be referred by peer educators or outreach workers (OR = 1.8, 95 % CI = 1.4-2.4) and more likely to be self-referred (OR = 1.8, 95 % CI = 1.3-2.4) or referred by relatives, friends, or colleagues (OR = 2.9 (1.7-4.9).

**Table 4:** Comparisons of HIV testing and counseling among FEWs at midterm (*n* = 450) and endpoint (*n* = 556)

HIV testing and counselling	Midterm	Endpoint	
	*n* (%)	*n* (%)	OR (95 % CI)
Tested for HIV in the past 6 months	334 (74.2)	311 (63.9)	1.3 (1.1-1.6)
Facility where most recent test was performed			
Public health center/clinic/hospital	61 (17.7)	68 (13.8)	1.3 (0.9-1.9)
Government or NGO VCCT	96 (27.8)	41 (8.4)	3.3 (2.3-4.9)
Private hospital/clinic/laboratory	42 (12.2)	112 (22.8)	1.9 (1.3-2.7)
C/PITC	144 (41.7)	252 (51.3)	1.7 (1.4-2.2)
Other	2 (0.6)	18 (3.7)	6.3 (1.5-27.4)
Persons who referred you for the most recent HIV testing			
Myself	85 (24.6)	226 (46.2)	1.9 (1.4-2.5)
Relatives/friends/colleagues	19 (5.5)	79 (16.1)	2.9 (1.7-4.9)
Peer educator/outreach workers/NGO staff	210 (60.9)	162 (33.1)	1.8 (1.4-2.4)
Other (partner, relative, manager)	31 (9.0)	22 (2.6)	2.0 (1.1-3.5)
Received the most recent HIV test result	326 (97.6)	484 (98.6)	1.1 (0.9-1.3)
Received counseling for the most recent HIV test	294 (88.0)	423 (87.6)	1.1 (0.8-1.2)

Abbreviations: *C/PITC* community/peer-initiated testing and counseling, *FEWs*, female entertainment workers, *NGO* non-governmental organization, *OR* odds ratio, *VCCT* voluntary confidential counseling and testing

Values are number (%) with OR (95 % CI) for categorical variables and mean ± SD with p-value for continuous variables

### Family planning and reproductive health

Table 5 shows the comparisons of family planning and reproductive health among women at midterm and endpoint. Among women who were sexually active, only 31.6 % of women at midterm reported currently using a contraceptive method compared to 45.5 % among women at endpoint (OR = 1.4, 95 % CI = 1.1-1.8). Regarding types of the method being used, women at endpoint were significantly less likely to report using uncommon methods such as intrauterine device, implant, calendar, or natural ways (OR = 3.5, 95 % CI = 1.8-6.5). Women at midterm were significantly more likely to report having at least one induced abortion in lifetime (OR = 2.0, 95 % CI = 1.5-2.6) and during the time working as an FEW (OR = 1.4, 95 % CI = 1.1-1.7). Regarding the facility of choice, women at endpoint were significantly less likely to report receiving the most recent abortion services at a public health facility compared to women at midterm (OR = 5.8, 95 % CI = 1.7-19.6).

**Table 5:** Comparisons of SRH and care seeking behaviors among FEWs at midterm (*n* = 450) and endpoint (*n* = 556)

Sexual reproductive health	Midterm	Endpoint	
	*n* (%)	*n* (%)	OR (95 % CI)
Currently using a contraceptive method	142 (31.6)	253 (45.5)	1.4 (1.1-1.8)
Type of contraceptive method being used			
Pills	24 (16.7)	64 (25.6)	1.5 (0.9-2.5)
Condom	63 (44.7)	106 (42.4)	1.1 (0.7-1.5)
Injection	6 (4.2)	22 (8.8)	2.1 (0.8-5.2)
Withdrawal	18 (12.4)	43 (17.0)	1.3 (0.7-2.4)
Other (IUD, implant, calendar, natural ways)	31 (21.7)	16 (6.2)	3.5 (1.8-6.5)
Mean number of pregnancies in lifetime	2.0 ± 2.6	1.5 ± 2.4	0.26
Mean number of pregnancy during working as FEW	0.6 ± 1.1	0.7 ± 1.6	0.45
Had at least one induced abortion in lifetime	195 (43.3)	122 (21.9)	2.0 (1.5-2.6)
Had at least one abortion during working as FEW	122 (27.1)	119 (21.4)	1.4 (1.1-1.7)
More than one abortion during working as an FEW	42 (9.3)	55 (9.9)	1.1 (0.7-1.6)
Place where most the most recent abortion was performed			
Public health center/clinic/hospital	28 (14.3)	3 (2.5)	5.8 (1.7-19.6)
Private clinic /hospital	90 (45.9)	56 (45.2)	1.1 (0.7-1.5)
Pharmacy (self-abortion)	60 (30.8)	49 (40.2)	1.3 (0.8-2.0)
NGO clinic/hospital	13 (6.6)	12 (9.7)	1.5 (0.6-3.3)
Other (including traditional birth attendant)	5 (2.6)	4 (3.2)	1.3 (0.3-4.8)

Abbreviations: *CI* confidence interval, *FEWs* female entertainment workers, *IUD* intra uterine device, *NGO* non-governmental organization, *OR* odds ratio

Values are number (%) with OR (95 % CI) for categorical variables and mean ± SD with p-value for continuous variables

## Discussion

We found several positive changes in key outcome indicators among women at midterm and endpoint, including a reduction in the prevalence of STI symptoms and induced abortion rates and an increase in the likelihood of seeking care and treatment for STI symptoms as well as contraceptive use. We attribute these findings to the combination of community-based peer outreach programs and integrated HIV and SRH services. However, several unfavourable findings, such as the steady decrease in consistent condom use with commercial sex partners, merit attention. Moreover, the prevalence rate of consistent condom use with unpaid or regular partners remained very low and did not change appreciably over the project lifespan.

A possible explanation for the decrease in consistent condom use with commercial sexual partners is that the average number of clients that women reported being sexually active with in the past three months decreased from 5.5 among women at midterm to 3.6 among women at endpoint, and almost 80 % of women at endpoint who were involved in commercial sexual activities reported only having 0–2 clients in the past three months. This association between number of clients and consistency of condom use is supported by others studies of sex workers in other developing countries. A study in Ghana found that female sex workers with more than seven clients per month were more likely to report consistent condom use than those with seven or fewer clients [25]. Similarly, another study in Uganda found that female sex workers with more than 10 clients in the past month were more likely to report consistent condom use than female sex workers with fewer than 10 clients [26].

In addition, there was a slow-but-steady decline in the prevalence rate of consistent condom use when having sexual intercourse with unpaid or regular partners reported by women from midterm to endpoint. The possible explanation of the low condom use among unpaid or regular partners can be similar to condom use with commercial sexual partners where there is a trustworthy emotional bond making women feel they are at lower risk for transmission of HIV and STIs. These findings strengthen the theory that value of protecting oneself with a condom decreases when women feel they are able to trust the person they have sexual intercourse with such as a significant other or husband. A study conducted in Cambodia in 2003 found that the most common reason why female sex workers reported not using condom with their non-paying, romantic partners was that they loved them [27]. Additional studies support the association between increased trust and lower condom use between female sex workers and their boyfriends in Kenya [28, 29].

From midterm to endpoint, there was a decrease in percentage of STI symptoms reported in addition to an increase in the proportion of women who sought care and treatment for their STI symptoms. The dramatic increase by 20 % for the amount of women who reported seeking care for the symptoms from midterm to endpoint may be attributed to the increased outreach sessions and the community health volunteer activities. These types of community-based activities have been shown to increase rates of STI screening and treatment among female sex workers in other settings [30]. In addition, the endpoint results demonstrate safer practices of healthcare seeking behaviors compared to previous years, where there was a noteworthy increase in the proportion of women who visited an NGO clinic as the first place to receive screening and treatment for their most recent STI symptoms and a significant decrease in the proportion of women seeking treatment from a pharmacy for the symptoms. This may also be a result of improved information dissemination through outreach sessions about safe healthcare-seeking practices.

A significant decrease in the number of women who had experienced at least one induced abortion during the time working as a FEW was shown from midterm to endpoint. However, the facilities where women visited to seek abortion services remained concerning. Among women at both midterm and endpoint, fewer FEWs reported going to public facilities, the safest options, and instead private clinics were the most common place where women sought abortion services, followed by a large portion of women who experienced self-abortion by taking medications from a pharmacy. Among women who had an abortion during the time working as a FEW, 63.5 % had received SRH education in the past six months, and 12 % answered “no” or “don’t know” when asked if they were able to find condom when they needed it.

The decrease in consistent condom use and in the proportion of women who had received some form of HIV or SRH education may be attributed to challenges in program implementation. One of KHANA’s demonstration centers for providing comprehensive and quality healthcare services for FEWs and their partners, the Purple House, closed in 2013 due to deficiency in funding support. The Purple House provided education sessions on HIV, SRH, family planning, condom negotiation, human rights, and gender-based violence; and linking women to health services including VCCT, STI care and treatment, and SRH care. The impact of the SAHACOM in the second half of the project life could have been attenuated by the closure of such an important facility.

Another difficulty in reaching the SAHACOM’s life project goals is the frequent mobility of the target populations. In both midterm and endpoint data, many FEWs reported not staying at the current place of work very long (under a year). This high mobility can make it difficult to follow-up and can be challenging for outreach workers and community support volunteers to provide HIV education, VCCT, and referral support to healthcare services. Both the closure of support centers and high mobility rates of FEWs demonstrate the necessity of quality work from community support volunteers and outreach workers to reach and address the needs of the women.

This study has several limitations. First, findings from this study might be limited by unknown reliability and validity of the tools. However, the questionnaire was developed using items adapted from previous studies in the same population, carefully reviewed by experts in this area, and pretested before the final version was constructed. Second, baseline data were not collected, and comparisons of key outcome indicators were made using data from the midterm and the endpoint surveys. This condition made the measurements of changes from the baseline to the endpoint difficult. Third, as with any self-reported measures, there were inherent biases potential for both underreporting and over-reporting in the variables. Fourth, it is possible that recall bias was a factor as participants were asked to recall events that had taken place over the past several months. The final limitation concerns the fact that only behavioural data were collected which may not reflect the respondents’ actual risk for HIV or STIs.

## Conclusions

This impact evaluation of the SAHACOM demonstrates improvements in important sexual behaviors and healthcare-seeking indicators such as STI treatment, contraceptive use, and induced abortion among FEWs which may be attributed to the unique community-based and peer-led design of the HIV/SRH integrated program. However, programmatic changes need to be taken into consideration in order to successfully increase the prevalence rates of consistent condom use, particularly with unpaid or regular partners and improving access to safe abortion services at public or NGO facilities.

## Abbreviations

AIDS: Acquired immunodeficiency syndrome
ART: Antiretroviral therapy
CI: Confidence interval
C/PITC: Community/peer-initiated testing and counselling
HIV: Human immunodeficiency virus
IUD: Intra uterine devices
MDG: Millennium development goals
NCHADS: National Center for HIV/AIDS, Dermatology, and STD
NGO: Non-governmental organization
OR: Odds ratio
SAHACOM: Sustainable Action against HIV and AIDS in Communities
SD: Standard deviation
SRH: Sexual reproductive health
STIs: Sexually transmitted infections
VCCT: Voluntary confidential counseling and testing
